# Integration of Small RNA and Transcriptome Sequencing Reveal the Roles of miR395 and ATP Sulfurylase in Developing Seeds of Chinese Kale

**DOI:** 10.3389/fpls.2021.778848

**Published:** 2022-02-03

**Authors:** Weiling Tang, Yijiao Zhao, Jiajing Zeng, Zunwen Li, Zhenlin Fu, Mengyu Yang, Donglin Zeng, Xiaodong Chen, Zhongxiong Lai, Gefu Wang-Pruski, Rongfang Guo

**Affiliations:** ^1^College of Horticulture, Institute of Horticultural Biotechnology, Fujian Agriculture and Forestry University, Fuzhou, China; ^2^Joint FAFU-Dalhousie Lab, College of Horticulture, Fujian Agriculture and Forestry University, Fuzhou, China; ^3^Department of Plant, Food, and Environmental Sciences, Faculty of Agriculture, Dalhousie University, Truro, NS, Canada

**Keywords:** miR395, APS3, miRNA, seed development, miR156

## Abstract

Seed development is closely related to plant production and reproduction, and MicroRNAs (miRNA) is widely involved in plant development including seed development. Chinese kale, as a Brassicaceae vegetable, mainly depends on seed for proper reproduction. In the present study, Chinese kale seed and silique at different stages were selected to establish small RNA (sRNA) libraries including silique wall sRNA libraries at torpedo-embryo stage (PC), silique wall sRNA libraries at cotyledonary-embryo stage (PD), seed sRNA libraries at torpedo-embryo stage (SC), and seed sRNA libraries at cotyledonary-embryo stage (SD). The results showed that miRNA expressed differentially in the seeds and corresponding siliques at different stages. To further clarify the functional mode of miRNA in the process of seed development, Kyoto encyclopedia of genes and genomes (KEGG) enrichment analysis was performed on target genes of the differentially expressed miRNAs, and these target genes were mainly enriched in plant hormone signal transduction, primary and secondary metabolic pathways. After joint analysis with the transcriptome change of the corresponding period, miR156-*SPL10*/*SPL11*, miR395-*APS3*, and miR397-*LAC2*/*LAC11* modules were identified to be directly involved in the development of Chinese kale seeds. What’s more, modified 5′RLM-RACE and *Agrobacteria*-mediated Chinese kale transient transformation suggest miR395b_2 is involved in sulfur metabolism during seed development by regulating its target gene *APS3*.

## Introduction

Chinese kale, a kind of Brassicaceae annual or biennial vegetable crop, is mainly propagated by seeds (Guo et al., 2019). The normal development of seed can ensure the smooth accumulation of dry matter, which can be used for seed germination. Healthy seeds are key to obtaining robust seedlings, which lay the foundation for the high quality and yield of crops. The development of seed includes the development of an embryo and the accumulation of storage materials in Brassicaceae vegetables.

MicroRNAs (miRNAs) are a group of small endogenous non-coding RNAs of 21∼24 nucleotides (nt) in eukaryotes with regulatory functions. Plant miRNAs regulate the expression of target genes by either transcript cleavage or translational inhibition. As an important post-transcriptional regulator of plant development and seed formation, miRNAs participate in seed development by regulating hormone signaling, starch synthesis, sugar conversion, and cell growth. Seed development-related miRNAs have been identified in monocot crops *Oryza sativa* (Zhang et al., 2013; Duan et al., 2016; Pan et al., 2018; Miao et al., 2019), *Triticum aestivum* (Han et al., 2014), and *Zea mays* (Kang et al., 2012) as well as dicots *Arabidopsis thaliana* (Reyes and Chua, 2007; Nodine and Bartel, 2010; Takanashi et al., 2018), *Arachis hypogaea* (Ma et al., 2018), and *Brassica napus* (Huang et al., 2013). In monocotyledonous plant, OsmiR156 targets *IPA1* (Ideal Plant Architecture 1) and regulates grain yield and seed dormancy in rice (Miao et al., 2019), while OsmiR396-*GRF4* (Growth Regulating Factor 4) and OsmiR397-*LAC* (Laccase) can control grain-size and yield (Zhang et al., 2013; Duan et al., 2016). In dicotyledonous plants, AtmiR156 targets *SPL10* and *SPL11* (Squamosa Promoter Binding Protein-like) and regulates cell division and differentiation during seed embryo development (Nodine and Bartel, 2010). Plants expressing the AtmiR160-resistant *ARF17* (Auxin Response Factor) may cause abnormal embryo symmetry (Mallory et al., 2005). AtmiR167-mediated repression of *ARF8* is required in embryogenesis (Plotnikova et al., 2019). AtmiR393-*TIR1* (Transport Inhibitor Resistant 1) is related to the maturity of *Arabidopsis* seeds (Xu, 2016).

Accompanied with the seed development, the metabolites in the seed were changed a lot. Regulation of metabolism direction and metabolites content has been demonstrated in Arabidopsis. For example, the accumulation of anthocyanins was changed in the plants overexpressing AtmiR156 (Wang et al., 2020), the glucosinolate biosynthesis was affected by the expression of AtmiR826 upon nitrogen starvation (Liang et al., 2017), miR395 is significantly up-regulated during sulfate limitation (Kawashima et al., 2011) and the over-expression of AtmiR395 results in sulfur deficiency symptoms and accumulated more sulfate in the shoots (Liang et al., 2010).

Chinese kale is an important Brassica vegetable with abundant glucosinolates. The glucosinolates content was highest in the reproductive part, especially the seed. We have previously identified two key stages of glucosinolate accumulation in Chinese kale seed, that is, torpedo-embryo stage and early cotyledonary embryo stage. During the transition from torpedo- to the early cotyledonary-embryo stage, the glucosinolate content changed significantly (Zhao et al., 2021). To understand the role of miRNA in seed development and glucosinolate metabolism, the seed and corresponding silique wall at different developmental stages (torpedo- and early cotyledonary-embryo stage, respectively) were selected to construct small RNA (sRNA) libraries. The miRNA in the seed and silique wall of Chinese kale was identified via small RNA sequencing. Subsequently, by the means of integrative small RNA and transcriptome sequencing, differentially expressed miRNAs and their target genes were analyzed in the current study. Finally, modified 5′RLM RACE was used to identify the cleavage site between miRNA and its target mRNA. Furthermore, we established a new *Agrobacterium*-mediated transient transformation system at Chinese kale cotyledons, which provided a new platform for functional characterization of Chinese kale genes.

## Materials and Methods

### Plant Material

Seeds of Chinese kale (*Brassica oleracea cv* HuangHua) were purchased from Gaoda seed shop (Fuzhou, China). The cultivation of Chinese kale has followed the method as described in Zhao et al. (2021). When the flowers were fully expanded, the stage of embryo in seeds was observed under a microscope (Olympus IX73, Japan), the seeds and silique walls under torpedo- and early cotyledonary-embryo stages were collected separately. The samples were quickly frozen in the liquid nitrogen and stored in a –80°C refrigerator for the following measurements.

### Total RNA Extraction and sRNA Library Construction

The RNA of seeds and seed silique walls were extracted using Trizol reagent (Invitrogen, Carlsbad, CA, United States) from Chinese kale at the torpedo- and early cotyledonary-embryo stages and repeated three times, respectively. The purity of the samples was measured using a NanoDrop 1000 spectrophotometer (Thermo Fisher Scientific, Wilmington, DE, United States) and the concentration of the RNA samples was measured by using a Qubit ^®^ 2.0 fluorometer (Life Technologies, CA, United States).

Before library construction, RNA samples after isolation were additionally treated with DNAase. We examined the integrity of the extracted RNA by 1% agarose gel electrophoresis. The A260/A280 ratios of RNA extracted ranged from 1.8 to 2.1. Agarose gel electrophoresis showed clear 28S rRNA and 18S rRNA bands, and the brightness of 28S rRNA bands was approximate twice the 18S rRNA. Twelve small RNA libraries were constructed by the small RNA Sample Pre Kit (Illumina, San Diego, CA, United States). Agilent 2100 Bioanalyzer (Agilent, Palo Alto, CA, United States) and ABI StepOnePlus Real-Time PCR System (Thermo Fisher Scientific, Waltham, MA, United States) were used to measure the quality and quantity of the constructed libraries. HiSeq2500 (MGI Tech Co., Ltd., China) was used for high-throughput sequencing with a read length of single-end 50 nt. The related databased can be found in SRA RJNA770111^1^ and SRA PRJNA770465^2^.

### Classification and Annotation of sRNA

To identify the potential conserved and novel miRNAs of Chinese kale, a bioinformatics analysis of sRNA sequencing was conducted. Firstly, low-quality tags and contaminants were discarded. Secondly, using Bowtie2 software (Langmead and Salzberg, 2012) to compare the sequence of clean reads with miRbase (Kozomara and Griffithsjones, 2014) and Rfam database (Nawrocki et al., 2015) to filter ribosomal RNA (rRNA), transfer RNA (tRNA), small nuclear RNA (snRNA), small nucleolar RNA (snoRNA), other coding RNA and repetitive sequences. The unique sRNA sequences were used to search for miRNA sequences using miRBase 21 to identify the conserved miRNAs in Chinese kale. Finally, the novel miRNAs from the surplus unannotated small RNAs were predicted by Mireap^3^. sRNA was annotated according to the priority order of MiRbase > pirnabank > snoRNA(human/plant) > Rfam > other sRNA.

### Analysis of Differentially Expressed MicroRNAs and Genes

Counting the expression level of miRNAs in seed sRNA libraries at torpedo- (SC) and cotyledonary-embryo stage (SD), as well as silique wall sRNA libraries at torpedo- (PC), and cotyledonary-embryo stage (PD), which were normalized by TPM algorithm (Hoen et al., 2008). DEGseq (Wang et al., 2010) was used to obtain differentially expressed miRNAs between PC and PD, PC and SC, PD and SD, SC and SD sRNA libraries. With | log2 (FC) | ≥ 1, FDR (False Discovery Rate) ≤ 0.01 as the screening criterion for differentially expressed miRNA. FC is the ratio of miRNA expression in different sRNA libraries.

DEGseq was used for differential expression genes analysis between groups, and FPKM was used to analyze the expression level of DEGs, and the Benjamini–Hochberg method was used to correct the significant *P*-values. Finally the corrected *P*-value, that is, *Q* value ≤ 0.001, | log2(fold change)| ≥ 1 as a screening criterion for the significance of differentially expressed genes.

### Target Gene Prediction and Annotation of Differentially Expressed MicroRNAs

TAPIR and TargetFinder (Fahlgren and Carrington, 2010) were used to predict the target genes of conserved and novel miRNAs. Then select the intersection as the prediction result. Using BLAST software (Altschul et al., 1997) to compare the predicted target genes sequence with the gene ontology (GO) (Ashburner et al., 2000), Kyoto encyclopedia of genes and genomes (KEGG) databases (Kanehisa et al., 2007) to obtain annotation information of the target genes.

### *Agrobacterium*-Mediated Chinese Kale Transient Transformation

The Chinese kale was cultivated in the plug for 2 weeks and the cotyledons were used as a transient transformation material. The pre-miR395b_2 was amplified by PCR from Chinese kale cDNA, then the PCR product was cloned into the binary vector *pCAMBIA1302* (mgfp5 Hygromycin B/Kanamycin Resistant Plant Expression Vector) by homologous recombination kit (Yeasen 10911ES20, China). The correct recombinant vector was introduced into *Agrobacterium* GV3101 for transformation. Pick *Agrobacterium* from a single colony, using a sterile toothpick, place it inside a sterile 50 mL centrifuge tube with 15 mL LB liquid media supplemented with the appropriate antibiotics, and culture them at 28°C and 180 rpm overnight. Spin down 50 mL centrifuge tube with *Agrobacterium* at 5,000 *g* for 10 min, discard LB medium supernatant, eliminate as much supernatant as possible. Resuspend with vortex the *Agrobacterium* deposit using 15 mL freshly prepared infiltration buffer (10 mM MES, 10 mM MgCl_2_⋅6H_2_O, 100 μM AS). After resuspension, leave cultures for 2 h in darkness at room temperature. Fill a 1 mL needleless syringe with the resuspended culture at a final OD 600 of 0.6, perform the infiltration by pressing the syringe (without needle) on the abaxial side of the Chinese kale cotyledon while exerting counter-pressure with a fingertip on the adaxial side (Wang, 2013). Sampling after injection at 0, 6, 12, 24, 48, and 72 h for subsequent real Time quantitative PCR (RT-qPCR) experiments.

### RT-qPCR Analysis

Using Primer Premier5 software to design the RT-qPCR primers for pre-miR395b_2 and its target genes *APS1-1*, *APS1-2*, *APS3*, and *APS4* (Table 1), with Actin as the internal reference gene. Total RNA was extracted using RNAiso Plus (Takara), Hieff III 1st Strand cDNA Synthesis SuperMix for qPCR (Yeasen) was used to reverse transcription of RNA into cDNA, and Hieff qPCR SYBR Green Master Mix (Yeasen) for RT- qPCR, CFX96 Real-Time PCR Detection System for RT-qPCR amplification.

**TABLE 1:** Primers that were used in this study.

Use	Primer	Sequence 5′→3′
Modified 5′RLM RACE	*APS3*-R1	CACCACCAATGAGCCAGTTT
	*APS3*-R2	GGTCGTGCCCCAAGTTCTA
	5′RACE Outer Primer	GCTGATGGCGATGAATGAACACTG
	5′RACE Inner Primer	CGCGGATCCGAACACTGCGTTTGC TGGCTTTGATG
RT-qPCR	*Actin*-F	CTGTGACAATGGTACCGGAATG
	*Actin*-R	ACAGCCCTGGGAGCATCA
	pre-miR395b_2-F	GAGAAGAAGCATTACCGAAACAG
	pre-miR395b_2-R	CACCTAGAGTCCCCCCAAAC
	*APS1-1*-F	GAGATTTACAAGCATCCCAAGG
	*APS1-1*-R	CAAAGACAGCATCCGCACC
	*APS1-2*-F	AAGGCTGATGATGTTCCTCTAAGT
	*APS1-2*-R	CTTCGGTTGGACCAGCATAA
	*APS3*-F	TGCCGAGAGTGAAACTGACG
	*APS3*-R	CGATGGCGAGAACGATAGG
	*APS4*-F	CGCCTCAACATTCTTCCCTT
	*APS4*-R	CCAACACTTTCCAACCAGAGG
PCR	pre-miR395b_2-IF	GGACTCTTGACCATGGATGTCCCCT TGAGTTCCCTGAAAC
	pre-miR395b_2-IR	CTTCTCCTTTACTAGTCACCTAGAG TCCCCCCAAACAC
Colony PCR	35S-F	GTGGATTGATGTGATATCTCC
	GFP-R	CTGACAGAAAATTTGTGCCC

### Modified 5′RLM RACE

Predict the cleavage site of miR395 and its target gene ATP sulfurylase (APS) by psRNATarget online software. Primer Premier5 software was used to design primers (Table 1), and the modified 5′RLM-RACE method was used to verify the cleavage site. Modified 5′RLM-RACE was carried out using the first choice RLM-RACE Kit as recommended by the manufacturer (ThermoFisher). Nested PCR requires two sets of primers which are used to amplify a specific DNA fragment using two separate runs of PCR (outer PCR and inner PCR). The inner PCR products were cloned to the 18-T vector (Takara) and subjected to sequencing analysis.

### Statistical Analysis

Statistical analysis was performed using SPSS26.0 software. Pathway builder tool software version 2.0 was applied to build the regulatory network, a heatmap was obtained by using TBTools (Chen et al., 2020).

## Results

### Establishment of sRNA Databases of the Seed and Corresponding Silique Wall at Different Stages

**FIGURE 1:**
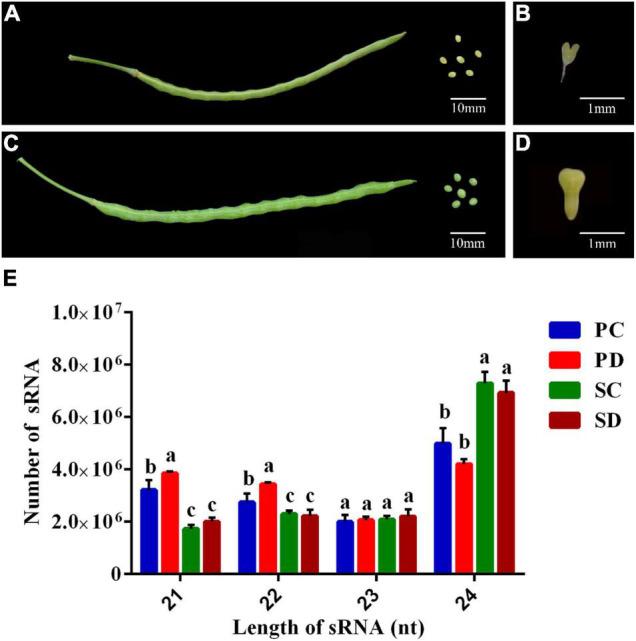
Phenotypes of siliques, seeds, and embryos at torpedo- and early cotyledonary-embryo stages of Chinese kale and the sRNA length distribution of different libraries. **(A)** Phenotype of siliques and the corresponding seeds at the torpedo-embryo stage of Chinese kale. The scale bar represents 10 millimeters (mm). **(B)** Torpedo embryo in siliques at **(A)**. The scale bar represents 1 mm. **(C)** Phenotypes of siliques and the corresponding seeds at the cotyledonary-embryo stage of Chinese kale. The scale bar represents 10 mm. **(D)** Early cotyledonary-embryo in siliques at **(C)**. The scale bar represents 1 mm. **(E)** Length distribution of sRNA in PC, PD, SC, and SD. The *X*-axis represented the lengths of sRNA ranging from 21 to 24 nucleotides (nt). The *Y*-axis is the number of sRNA at a specific length. Error bars represent the SE of three biological replicates. The differences labeled with distinct letters were considered significant at *p* < 0.05. PC and SC mean sRNA libraries of silique walls and seeds at the torpedo-embryo stage, respectively. PD and SD mean sRNA libraries of silique walls and seeds at the cotyledonary-embryo stage, respectively.

We have previously noticed that the glucosinolate content in Chinese kale increased significantly during the seed transition from the torpedo- to the early cotyledonary-embryo stage. To identify miRNAs involved in the development of Chinese kale seed, seeds and the corresponding silique walls at the torpedo-embryo (Figures 1A,B) and early cotyledonary-embryo stages (Figures 1C,D) were subjected to the high-throughput sequencing, respectively. Twelve sRNA libraries including the seeds and corresponding silique walls at the torpedo-embryo stage (SC and PC, respectively) as well as the seeds and corresponding siliques at the early cotyledonary-embryo stage (SD and PD, respectively) were constructed (Table 2). After removing low-quality reads, contaminants, and adaptors, the clean reads of SC, PC, SD, and PD were obtained and accounted for more than 92.00%. The Q20 value of the clean reads was greater than 99.80%, indicating that high-quality sRNAs were obtained by high-throughput sequencing and can be used for the following analysis. The clean reads were compared with the known sRNA database (miRBase, Rfam, siRNA, piRNA, and snoRNA). The sequences from the constructed libraries matched more than 75.00% with the genome sequences.

**TABLE 2:** The high-throughput sequencing data of 12 small RNA libraries with seeds and siliques at the torpedo-embryo and the early cotyledon-embryo stages.

Sample name	Raw tag	Clean tag	Percentage of clean tag (%)	Mapped tag	Percentage of mapped tag (%)
PC1	15,346,483	14,428,519	94.02	11,257,848	78.02
PC2	17,754,314	16,780,323	94.51	14,050,908	83.73
PC3	18,503,931	17,678,582	95.54	13,769,000	77.89
PD1	17,786,925	16,895,610	94.99	12,704,902	75.20
PD2	18,407,038	17,471,944	94.92	13,792,707	78.94
PD3	17,294,674	16,440,597	95.06	12,547,808	76.32
SC1	16,886,608	16,067,096	95.15	13,753,248	85.60
SC2	15,977,428	15,271,237	95.58	13,064,358	85.55
SC3	17,368,020	16,470,754	94.83	13,859,628	84.15
SD1	16,964,305	15,916,030	93.82	13,667,705	85.87
SD2	16,808,198	15,476,690	92.08	13,349,211	86.25
SD3	18,405,473	17,440,052	94.75	14,714,461	84.37

### The Size Distribution of sRNAs and First Base Nucleotide Bias of Known MicroRNAs in the Seed and Silique Wall Libraries

The length distribution of sRNAs in the seed and silique wall libraries was shown in Figure 1E. The sRNA length in the seeds and silique walls ranges from 18 nt to 30 nt and the 24 nt sRNA was the most abundant type, accounting for 45.88 and 42.68% in SC and SD, respectively, and 30.79 and 24.98% in PC and PD, respectively. The proportion of sRNA at different lengths varied in the seed and silique. The silique contained much more 21-nt sRNA and less 24-nt sRNA than the seed. No difference was detected in the comparison of PC *vs*. PD and SC *vs*. SD.

**FIGURE 2:**
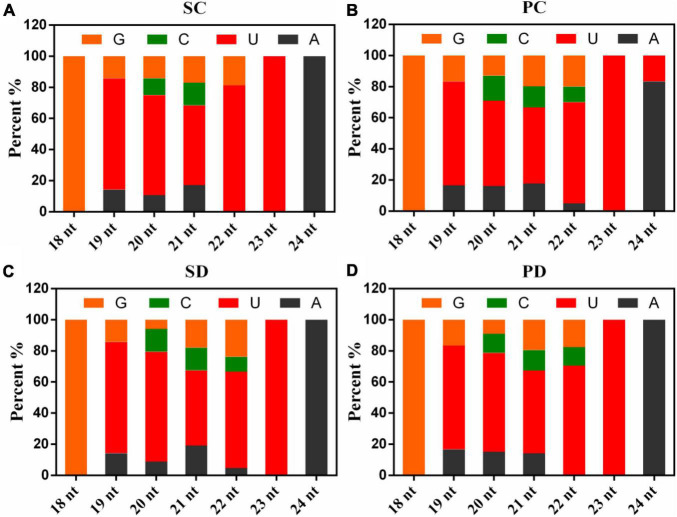
The first base distribution in PC, PD, SC, and SD. **(A)** The first base distribution in SC. **(B)** The first base distribution in PC. **(C)** The first base distribution in SD. **(D)** The first base distribution in PD. The *X*-axis represented the length of known miRNAs and the numbers on the histogram represent the number of miRNA species of that length. The *Y*-axis is the percent of the first nucleotide numbers at each length of known miRNAs. PC and SC mean sRNA libraries of silique walls and seeds at the torpedo-embryo stage, respectively. PD and SD mean sRNA libraries of silique walls and seeds at the cotyledonary-embryo stage, respectively.

As shown in Figure 2, analyses of the first nucleotide of the known miRNAs indicated that more than 45% of the miRNAs consisting of 19, 20, 21, 22, or 23 nt began with a Uridine, the 18-nt miRNAs started with a Guanine, while the 24-nt miRNAs started with an Adenine or a Uridine.

### Differentially Expressed MicroRNAs in the Seeds and Corresponding Silique at Different Stages

**FIGURE 3:**
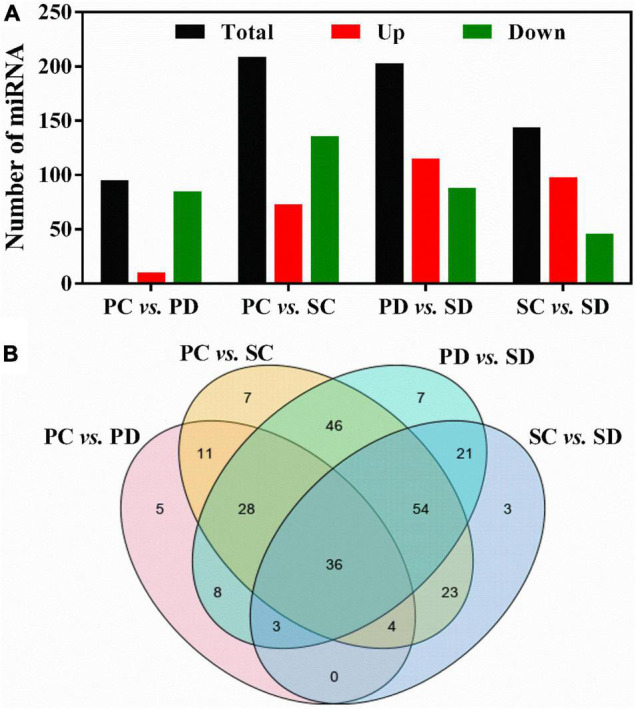
**(A)** Statistic of differentially expressed miRNA in PC *vs.* PD, PC *vs.* SC, PD *vs.* SD, and SC *vs.* SD. The *X*-axis represented comparative groups. The *Y*-axis represented the number of corresponding miRNAs. **(B)** Venn diagrams showing the number of common and specific differentially expressed miRNAs in comparisons of the four libraries. PC and SC mean sRNA libraries of silique walls and seeds at the torpedo-embryo stage, respectively. PD and SD mean sRNA libraries of silique walls and seeds at the cotyledonary-embryo stage, respectively.

By comparing the expression levels of miRNAs in PC *vs*. PD, PC *vs*. SC, PD *vs*. SD, and SC *vs*. SD, differentially expressed miRNAs were identified (Figure 3A). At the torpedo-embryo stage, there are 209 differentially expressed miRNAs in PC *vs*. SC, with 73 up-regulated miRNAs and 136 down-regulated miRNAs. At the cotyledonary embryo stage, 203 differentially expressed miRNAs were identified in the comparison of PD *vs*. SD, among these, 115 miRNAs were up-regulated and 36 miRNAs were down-regulated (Figure 3A). During the transition from torpedo- to the cotyledonary-embryo stage, 95/144 miRNAs were differentially expressed in the siliques and seeds respectively, among these, 10/98 miRNAs were up-regulated and 85/46 miRNAs were down-regulated (Figure 3A). Further analysis of the differentially expressed miRNAs revealed that 36 miRNAs were all differentially expressed in the four groups (Figure 3B). Five miRNAs were specific differentially expressed in PC *vs*. PD, seven miRNAs showed differential expression levels in PC *vs*. SC or PD *vs*. SD, and three miRNAs were only differentially expressed in SC *vs*. SD.

### GO and KEGG Analysis of Target Genes of Differentially Expressed MicroRNAs

**FIGURE 4:**
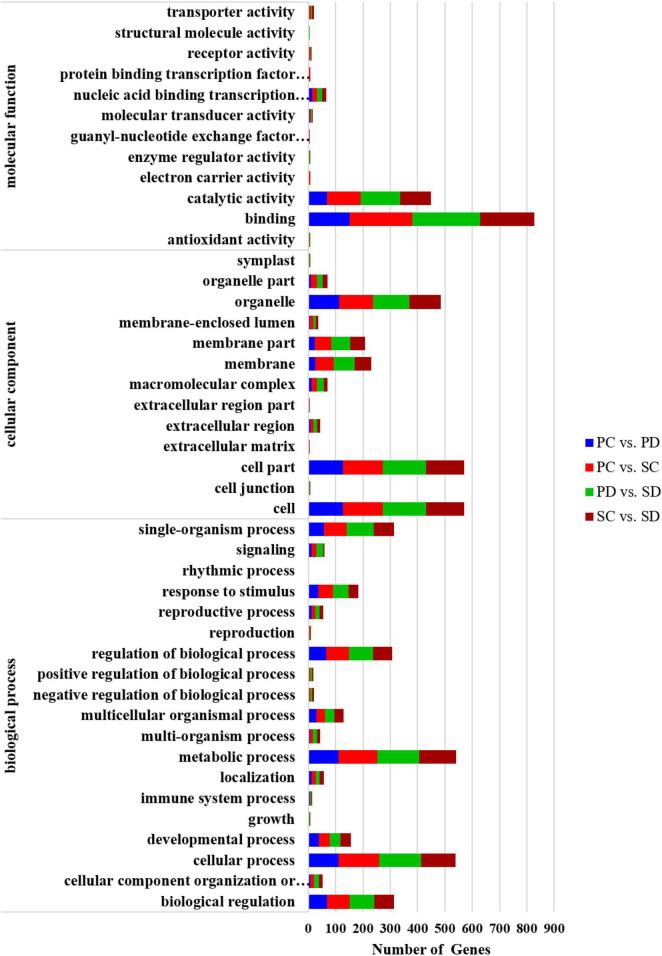
GO annotation of predicted target genes of differential miRNAs in PC *vs.* PD, PC *vs.* SC, PD *vs.* SD, and SC *vs.* SD. The *X*-axis represented the number of predicted target genes. The *Y*-axis is the GO term. PC and SC mean sRNA libraries of silique walls and seeds at the torpedo-embryo stage, respectively. PD and SD mean sRNA libraries of silique walls and seeds at the cotyledonary-embryo stage, respectively.

A total of 376, 611, 656, and 540 target genes were predicted for the differential miRNAs of PC *vs*. PD, PC *vs*. SC, PD *vs*. SD, and SC *vs*. SD, respectively. Gene annotations and classifications based on biological pathways (biological process: BP), cellular localizations (cellular component: CC), and molecular function (molecular function: MF) are provided in Figure 4. Nineteen classes of BPs were identified, with the two most frequent being “metabolic process” and “cellular process.” Among the MF categories, “binding” contained the most genes, followed by “catalytic activity.”

**FIGURE 5:**
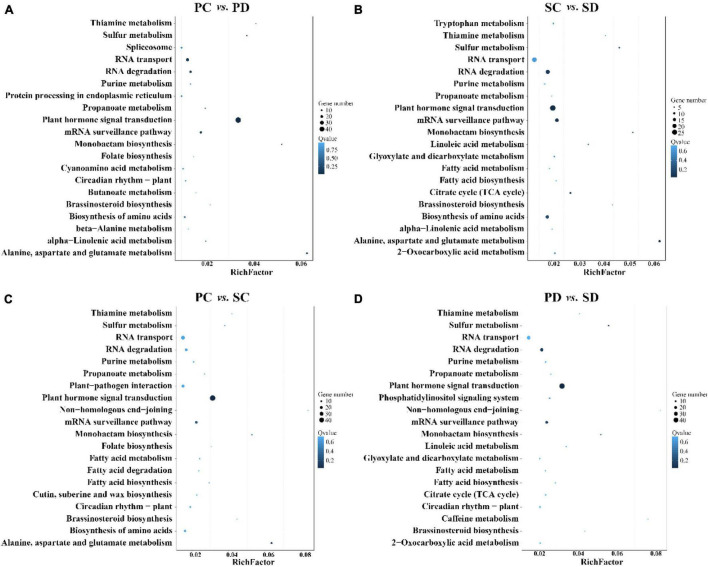
KEGG enrichment of differential miRNAs in PC *vs.* PD, SC *vs.* SD, PC *vs.* SC, and PD *vs.* SD. **(A)** KEGG enrichment of differential miRNAs in PC vs. PD. **(B)** KEGG enrichment of differential miRNAs in SC *vs.* SD. **(C)** KEGG enrichment of differential miRNAs in PC *vs.* SC. **(D)** KEGG enrichment of differential miRNAs in PD *vs.* SD. The *X*-axis is the Rich Factor (Rich Factor is calculated as candidate gene number in a specific term/total gene numbers) and the *Y*-axis represents the KEGG pathway, the size of the bubble indicates the number of genes annotated to a KEGG Pathway. The blue color represents the enriched *Q*-value. The darker the color, the smaller the *Q*-value. Showing the enrichment of the KEGG pathway in the form of a bar chart, and plotted the top 20 KEGG pathway with the smallest *Q*-value. PC and SC mean sRNA libraries of silique walls and seeds at the torpedo-embryo stage, respectively. PD and SD mean sRNA libraries of silique walls and seeds at the cotyledonary-embryo stage, respectively.

The KEGG pathway database was used to identify the biological pathways of target genes related to differentially expressed miRNAs in the four groups. The top 20 KEGG enrichment pathways are shown in Figure 5. The most enriched KEGG pathway in PC *vs.* PD, PC *vs.* SC, SC *vs.* SD, and PD *vs.* SD was the plant hormone signal transduction pathway. Furthermore, the significantly enriched 20 KEGG pathways in PC *vs.* PD, PC *vs.* SC, SC *vs.* SD, and PD *vs.* SD were general metabolic pathways and biosynthesis of secondary metabolites. It should be noted that the top 20 KEGG enrichment pathways in the four comparative groups all include the sulfur metabolism pathways. Further analysis revealed that genes enriched in the sulfur metabolism pathway are Bo3g081470 (*APS1-1*), Bo5g093400 (*APS1-2*), Bo1g057690 (*APS3*), and Bo7g065730 (*APS4*), all of these genes are targeted by miR395.

### Analysis of MicroRNAs Related to Seed Development

By referring to previous researches and the KEGG enrichment analysis, nine miRNA family members and their target genes were analyzed in this study. Pathway builder tool was applied to build the regulatory network of miRNAs and their target genes that may participate in the development of Chinese kale seed (Supplementary Figure 1). The results showed that *SPL10* and *SPL11* were regulated by miR156; *GAMYB* was regulated by miR159; *ARF10*, *ARF16*, and *ARF17* were regulated by miR160; *TIR1*, *AFB2*, and *AFB3* were regulated by miR393 and novel_mir91; β*G13-like* and β*G15-like* were regulated by miR319; *APS1*, *APS3*, *APS4*, and *SULTR2;1* were regulated by miR395; *GRF4* and β*G44* were regulated by miR396; *LAC2* and *LAC11* were regulated by miR397.

**FIGURE 6:**
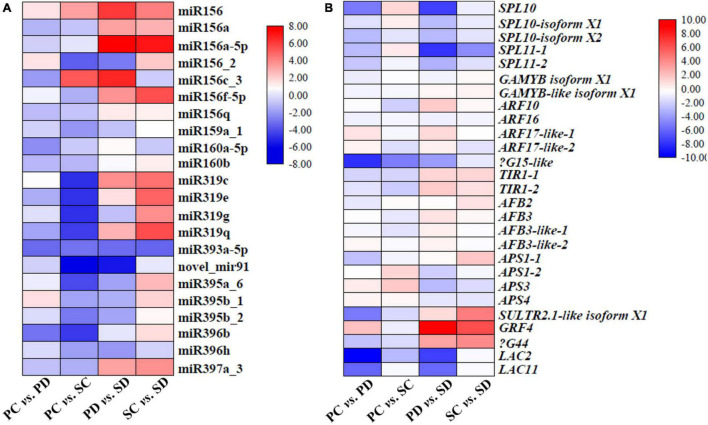
Heat maps represent the expression levels of miRNAs and their target genes related to seed development in PC *vs.* PD, PC *vs.* SC, PD *vs.* SD, and SC *vs.* SD. **(A)** The expression level of miRNAs that are related to seed development in PC *vs.* PD, PC *vs.* SC, PD *vs.* SD, and SC *vs.* SD. The *X*-axis represented different comparative groups. The *Y*-axis represented different miRNA family members. **(B)** Expression of predicted target genes of miRNAs in **(A)**. The *X*-axis represented different comparative groups. The *Y*-axis represented different predicted target genes. Different colors in the heat map represented different fold changes. PC and SC mean sRNA libraries of silique walls and seeds at the torpedo-embryo stage, respectively. PD and SD mean sRNA libraries of silique walls and seeds at the cotyledonary-embryo stage, respectively.

Combining sRNA high-throughput sequencing data and transcriptome sequencing data to analyze the expression level of the miRNA family members and their target genes (Figure 6). In PC *vs*. PD, excepts miR156c-2, miR156f-5p, miR319c, miR395a-6, and miR395b-1, the expression level of other miRNAs were down-regulated; in PC *vs.* SC, excepts miR156, miR156f-5p, and miR156c-3 expression were up-regulated, the expression of other miRNAs are down-regulated; in PD *vs*. SD, excepts miR156c_2, miR159_1, miR319g, miR393a-5p, novel_mir91, miR395a_6, miR395b_1, miR395b_2, and miR396h, the expression of other miRNAs were up-regulated; in SC *vs*. SD, excepts miR156c_3, miR160a-5p, miR393a-5p, and miR396h, the expression of other miRNAs was up-regulated (Figure 6A). In PC *vs*. PD, except *ARF17-1*, *ARF17-2*, *APS3*, and *APS4* expression level were up-regulated, the expression of other target genes were down-regulated; In PC *vs*. SC, excepts for the up-regulation of *SPL10*, *SPL10-isoform X1*, *APS1-2*, *APS3*, and *APS4*, the expression of other target genes were down-regulated; in PD *vs*. SD, excepts *ARF10*, *ARF17-1*, *TIR1-1*, *TIR1-2*, *AFB3*, *AFB3-like-1*, *AFB3-like-2*, *SULTR 2; 1-like isoform X1*, *GRF4*, and β*G44* expression were up-regulated, the expression of other target genes were all down-regulated; in SC *vs*. SD, except the up-regulation of *GAMYB-like isoform X1*, *TIR1-1*, *APS1-1*, *SULTR 2; 1-like isoform X1*, *GRF4*, and β*G44*, the expression of other target genes were down-regulated (Figure 6B).

### Analysis of the Expression Pattern of Pre-miR395b_2 and Its Target Genes in Chinese Kale

The target genes of differentially expressed miRNAs in the four comparative groups were mainly enriched in plant hormone signal transduction, metabolic pathways, and biosynthesis of secondary metabolites. Especially, *APS1-1*, *APS1-2*, *APS3*, and *APS4* are enriched in the sulfur metabolism pathway among the four comparative groups, which are targeted by differentially expressed miR395b_2, suggesting that miR395b_2 is involved in sulfur metabolism by regulating its target genes *APS* during seed development.

**FIGURE 7:**
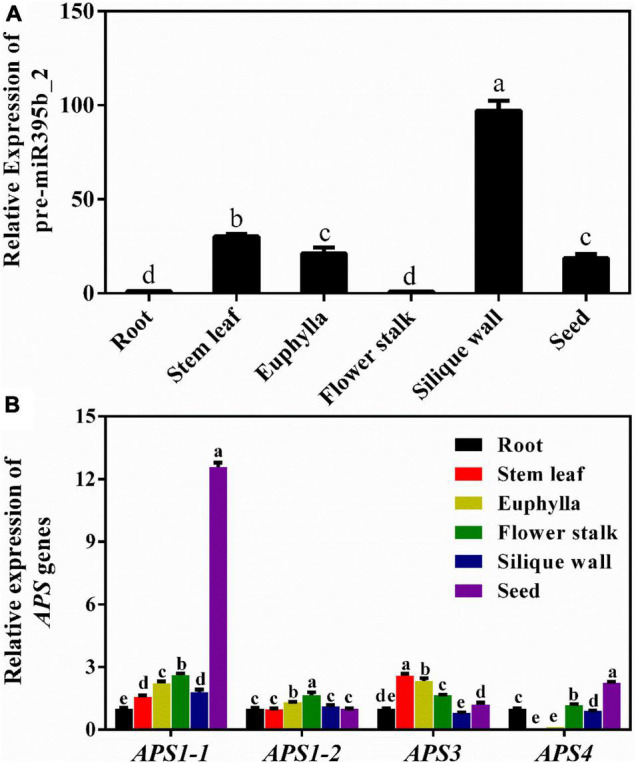
Expression patterns of pre-miR395b_2 and its target gene *APS* in different tissues of Chinese kale at the reproductive growth period. **(A)** RT-qPCR analysis of the expression of pre-miR395b_2 in the root, stem leaf, euphylla, flower stalk, silique wall, and seed at the reproductive growth period of Chinese kale. **(B)** RT-qPCR analysis of the expression levels of *APS1-1*, *APS1-2*, *APS3*, and *APS4* in the root, stem leaf, euphylla, flower stalk, silique wall, and seed at the reproductive growth period of Chinese kale. The different lowercase letters on the graph indicate significant differences among tissues at 0.05 level (*P* < 0.05). Error bars represent SE, *n* = 4.

Mature miRNA is formed by shearing and processing of pre-miRNA. Generally, the expression level of pre-miRNA is consistent with the expression level of mature miRNA. The relative expression level of pre-miR395b_2 and its target genes *APS1-1*, *APS1-2*, *APS3*, and *APS4* in different tissues during the reproductive growth period was detected by the RT-qPCR method (Figure 7). The results showed that the expression levels of pre-miR395b_2 varied in different tissues. Specifically, the expression level of pre-miR395b_2 is highest in the silique wall, while its transcripts are significantly low in root and flower stalk at the reproductive growth period (Figure 7A). The expression levels of *APS1-1* and *APS4* are highest in seed, the expression level of *APS1-2* is highest in flower stalk, and the expression level of *APS3* is highest in stem leaf (Figure 7B).

### Verification of miR395’s Target Gene APS by *Agrobacteria*-Mediated Transient Transformation Method

Cotyledons are the vegetative organs of Chinese kale, which store large amounts of nutrients and can provide sufficient raw materials for gene expression. Compared with true leaves, Chinese kale cotyledons have a soft tissue texture and fewer leaf veins, which is convenient for injection. In addition, Chinese kale is an unearthed plant from cotyledons, which can be used as research materials after seeds are cultivated for a few days. Based on the above advantages, we choose the cotyledons of Chinese kale as the research materials for the *Agrobacterium*-mediated transient transformation method.

**FIGURE 8:**
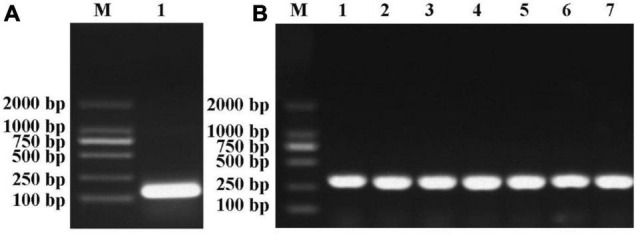
Pre-miR395b_2 cloning and colony PCR of *pCAMBIA1302-35S-pre-miR395b_2-GFP* recombinant vector. **(A)** The precursor of miR395b-2 from Chinese kale was cloned. Lanes from left to right are 2000-bp Marker and pre-miR395b_2 PCR product (Lane 1). **(B)** Colony PCR was used to identify the *pCAMBIA1302-35S-pre-miR395b_2-GFP* recombinant vector. Lanes from left to right are 2000-bp Marker and colony PCR product (Lanes 1–7).

To investigate the potential function of miR395, the precursors of miR395b_2 (99 bp) from Chinese kale was cloned (Figure 8A). The PCR product of pre-miR395b_2 was cloned into the *pCAMBIA1302* vector and introduced into DH5α competent cell, the colony PCR result showed that the seven single colonies picked at random were all positive (Figure 8B), which indicates that the *pCAMBIA1302-35S-pre-miR395b_2-GFP* recombinant vector was successfully constructed.

**FIGURE 9:**
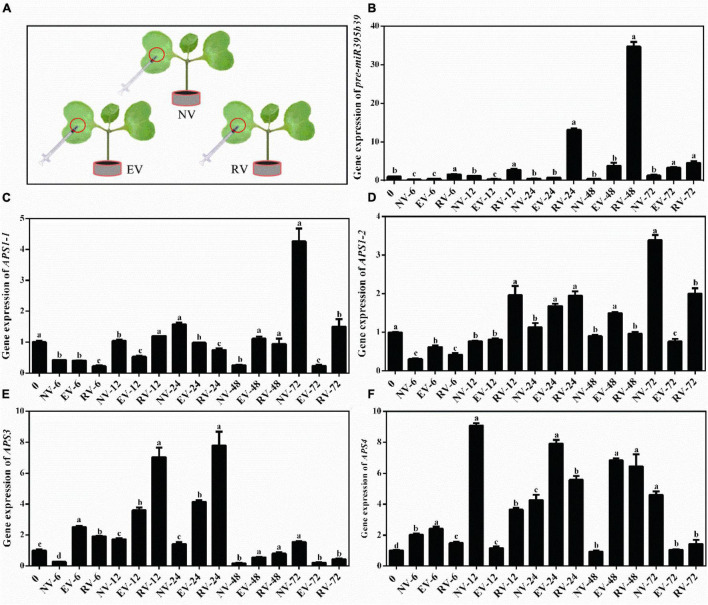
*Agrobacteria*-mediated Chinese kale transient transformation and RT-qPCR analysis of pre-miR395b_2 and *APS* genes in transgenic lines. **(A)** The resultant *Agrobacteria* was used to transient transform Chinese kale cotyledon through the injection method. NV represented the empty control, EV represented *pCAMBIA1302* empty vector, RV represented *pCAMBIA1302-35S-pre-miR395b_2-GFP* recombinant vector. RT-qPCR analysis of the expression of **(B)** pre-miR395b_2, **(C)** *APS1-1*, **(D)** *APS1-2*, **(E)** *APS3*, and **(F)** *APS4* in transgenic lines at different time points. The different lowercase letters on the graph indicate significant differences among tissues at 0.05 level (*P* < 0.05). Error bars represent SE, *n* = 4.

The resultant *Agrobacteria* with *pCAMBIA1302* empty vector and *pCAMBIA1302-35S-pre-miR395b_2-GFP* recombinant vector was used to transient transform Chinese kale cotyledon through the injection method, respectively (Figure 9A). Overexpression of pre-miR395b_2 was achieved by using Cauliflower mosaic virus (CaMV)35S to drive expression pre-miR395b_2 in Chinese kale. Using RT-qPCR, a higher accumulation of pre-miR395b_2 was observed in transgenic plants. From 0 to 48 h, compared to plants which injected no vector and injected with *Agrobacteria* harboring *pCAMBIA1302* empty vector, the expression level of pre-miR395b_2 has been increasing in transgenic plants which injected with *Agrobacteria* harboring *pCAMBIA1302-35S-pre-miR395b_2-GFP* recombinant vector. From 48 to 72 h, the expression level of pre-miR395b_2 is decreased (Figure 9B), suggesting that pre-miR395b_2 overexpression was successfully achieved in Chinese kale by *Agrobacterium*-mediated transient transformation. Meanwhile, we determined the expression level of *APS1-1* (Figure 9C), *APS1-2* (Figure 9D), *APS3* (Figure 9E), and *APS4* (Figure 9F), which are target genes of miR395b_2. From 0 to 24 h, the expression level of *APS3* has been increasing in transgenic plants in comparison with the untransformed controls plants and transformed *pCAMBIA1302* empty vector plants. From 24 to 72 h, on the contrary, the expression level of *APS3* has been decreased in the transgenic plants (Figure 9E). These results indicate overexpression of pre-miR395b_2 affects the expression levels of its target gene *APS3.*

### Modified 5′RLM RACE Verify the Cleavage Site Between miR395b_2 and *APS3*

**FIGURE 10:**
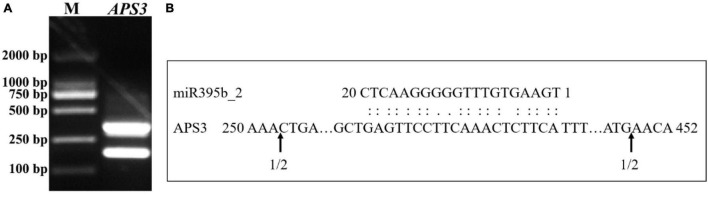
Experimental validation of the cleavage sites between miR395b_2 and *APS3*. **(A)** 5′RLM-RACE inner PCR of *APS3* of Chinese kale. **(B)** The cleavage sites between miR395b_2 and *APS3*. Partial *APS3* mRNA sequences were aligned with miR395b_2. Numbers indicate the fraction of cloned PCR products terminating at different positions.

In plants, miRNA functions via the cleavage of target genes at complementary sites, and the function mode of miRNA and target genes can be detected by using 5′RLM RACE. The cleavage site between miR395b_2 and *APS3* was validated by modified 5′RLM RACE assay, inner PCR agarose gel electrophoresis results showed two bands, which indicates there are two cleavage sites on *APS3* mRNA (Figure 10A). Sequencing analysis results suggest that the two cleavage sites on *APS3* mRNA were located outside of the complementary region (Figure 10B).

## Discussion

MicroRNAs function as key post-transcriptional factors regulating the expression of many genes related to seed development. Through high-throughput sequencing, the size distribution of sRNAs in Chinese kale was mainly distributed in the range from 21 to 24 nt. Target genes of differentially expressed miRNAs in Chinese kale seeds and silique walls at different stages were enriched in plant hormone signal transduction and metabolic pathways.

### MicroRNAs Related to Hormone Signal Transduction Participate in the Development of Seeds

Auxin is a key factor in seed development and participates in the processes of seed morphogenesis, cell division, and cell expansion (Schruff et al., 2005). It has been found that AtmiR160 regulates the expression of *ARF10*, *ARF16*, and *ARF17*, and plants expressing the AtmiR160-resistant *ARF17* may cause abnormal embryo symmetry (Mallory et al., 2005). The accumulation of *ARF16* resulting from the down-regulation of AhmiR160a might enhance the auxin response and thus enhance seed development in peanuts (Ma et al., 2018). In our study, the negative relationship of miR160 and *ARFs* was also noticed in different groups. Another module involved in the auxin regulation is miR393-*TIR1/AFBs*. It was reported that AtmiR393 regulates the expression of the auxin receptor *TIR1* and three other closely related auxin signaling F-box proteins (*AFB1*, *AFB2*, and *AFB3*) (Sunkar and Zhu, 2004). In this study, we identified that the expression of *AFB2*, *AFB3*, *AFB3-like-1*, and *AFB3-like-2* is negatively related to the transcripts abundance of miR393, which is consistent with previous studies that AtmiR393 affects plant sensitivity to auxin signals by negatively regulating the expression of *TIR1* (Chen et al., 2011) and AtmiR393-*TIR1*/*AFBs* is involved in the maturation of seed (Xu, 2016).

Laccase is the target gene of miR397, whose product is a laccase-like protein involved in brassinosteroid signal transduction (Zhang et al., 2013). OsmiR397 has been found to regulate *Oryza sativa* seed size and yield by targeting *LAC* to increase the susceptibility of plants to brassinosteroid. Overexpression of OsmiR397 can increase seed size, promote panicle branching, and increase the number of main spikelets, thereby increasing yield (Zhang et al., 2013). It was reported that AtmiR397b regulates lignin synthesis and seed yield by targeting *LAC4* (Wang et al., 2014). Consistent with these previous investigations, in this study, the expression of miR397-3 was higher in PD *vs.* SD and SC *vs.* SD, while the expression of *LAC2* and *LAC11* was lower in PD *vs.* SD and SC *vs.* SD, suggesting that miR397-3 target *LAC2* and *LAC11* to participate in the development of Chinese kale seed.

OsmiR159 is involved in gibberellin GA signal transduction by regulating *GAMYB* and *GAMYB-like* genes (Tsuji et al., 2006). Overexpression of AtmiR159 can correspondingly inhibit *GAMYB-like* (*MYB33* and *MYB101*) transcription, resulting in seed insensitivity to ABA signaling (Reyes and Chua, 2007). In *Oryza sativa*, the mRNA levels for *GAMYB* and *GAMYBL1* genes are negatively correlated with OsmiR159 levels during the anther development (Tsuji et al., 2006). In the present study, the expression of miR159a-1 was higher, while *GAMYB isoform X1* expression was lower in SD than in SC.

### Functions of miR395 by Targeting APS During Seed Development in Chinese Kale

Sulfur is one of the essential elements for plant growth and development, among which sulfate is the main form of sulfur in organisms. The target genes of AtmiR395 are the *ATP sulfurylase* gene (*AtAPS1*, *AtAPS3*, and *AtAPS4*) and sulfate transporter gene (*SULTR2; 1*), which catalyze the absorption of sulfuric acid inorganic salts, both of them play an important role in sulfur assimilation and transport (Jonesrhoades and Bartel, 2004; Allen et al., 2005). AtmiR395 enhances seed resistance to salt stress and drought stress by regulating the expression of *AtAPSs* (Kim et al., 2010). In our study, we observed that the expression of BomiR395b_2 was higher in SD than in SC, while the expression of *BoAPS1-2*, *BoAPS3*, and *BoAPS4* was lower in SD than in SC. indicating that and *BoAPS4*. What’s more, based on *Agrobacterium*-mediated Chinese kale transient transformation experiments, higher accumulation of pre-miR395b_2 was achieved in transgenic plants which overexpressed pre-miR395b_2 and then affected the expression level of its target gene *APS3.* This result demonstrated that BomiR395 was involved in the sulfur metabolism of seeds by negatively regulating *BoAPS3*. The subsequently modified 5’RLM RACE assay verified the two cleavage sites on *APS3* mRNA. However, the cleavage site was not following the prediction of psRNATarget online software. Regarding the dependence of bioinformatics on model plants, the cleavage site of miR395 on APS3 may be different in *Arabidopsis* and Chinese kale.

### MicroRNAs Related to Plant Cell Growth Were Abundant in Developing Seeds

miR156 is a type of miRNA that regulates cell growth by targeting and repressing the expression of the *SPL* gene family. AtmiR156 targets the *SPL10* and *SPL11* genes and regulates cell division and differentiation during Arabidopsis seed embryo development (Nodine and Bartel, 2010). Studies on Arabidopsis have found that when miRl56 expression is suppressed, its target genes *SPL10* and *SPL11* are prematurely expressed, and genes that are expressed later in the development of the seed are induced to express early (Nodine and Bartel, 2010). In this study, the results showed that the expression of miR156, miR156a, miR160a-5p, miR156-2, miR156f-5p, and miR156q was higher in SD than in SC, while *SPL10*, *SPL10-isoform X1*, *SPL10-isoform X2*, *SPL11-1*, and *SPL11-2* expression was lower in SD than in SC. This is consistent with the results of previous studies. OsmiR156 negatively regulates the expression of *SPL10* and *SPL11* (Nodine and Bartel, 2010). During *Brassica napus* seed development, BnmiR156 is mainly expressed in embryos and concentrated in cotyledons, and expression abundance gradually increases with seed maturity (Huang et al., 2013). This indicates that miR156 targets *SPL10* and *SPL11* and plays an important role in the development of Chinese kale seeds.

Besides, *GRF4* regulates grain size by affecting cell division and cell elongation in *Oryza sativa*. A study has found that miR396 regulates *GRF4* to control grain size and yield (Duan et al., 2016). In this study, *GRF4* was hardly expressed in PC, PD, SC, and SD by transcriptome data analysis.

## Conclusion

In this study, miRNAs and their target genes that are involved in the development of Chinese kale seeds were identified by high-throughput sequencing. During the transition from torpedo- to the early cotyledonary-embryo stage, hormone signal transduction (e.g., miR160-*ARF*, miR397-*LAC*) and metabolism (e.g., miR395-*APS*) related pathways were enriched. Moreover, we have demonstrated that miR395 is involved in sulfur metabolism by regulating its target genes *APS3*.

## Data Availability Statement

The datasets presented in this study can be found in online repositories. The names of the repository/repositories and accession number(s) can be found below: BioProject: PRJNA770111, PRJNA770465.

## Author Contributions

RG, XC, ZxL, and GW-P designed the research. WT, YZ, JZ, ZwL, ZF, MY, and DZ performed the research and wrote the manuscript. RG, WT, YZ, and JZ analyzed the data. All authors have read and approved the manuscript for publication.

## Conflict of Interest

The authors declare that the research was conducted in the absence of any commercial or financial relationships that could be construed as a potential conflict of interest.

## Publisher’s Note

All claims expressed in this article are solely those of the authors and do not necessarily represent those of their affiliated organizations, or those of the publisher, the editors and the reviewers. Any product that may be evaluated in this article, or claim that may be made by its manufacturer, is not guaranteed or endorsed by the publisher.

## Footnotes

1
https://submit.ncbi.nlm.nih.gov/subs/bioproject/SUB10406823

2
https://submit.ncbi.nlm.nih.gov/subs/bioproject/SUB10506456

3
https://sourceforge.net/projects/mireap/

## Supplementary Material

Supplementary Figure 1 Regulatory network of miRNAs and their predicted target genes related to Chinese kale seed development in PC *vs.* PD, PC *vs.* SC, PD *vs.* SD, and SC *vs.* SD. PC and SC mean sRNA libraries of silique walls and seeds at the torpedo-embryo stage, respectively. PD and SD mean sRNA libraries of silique walls and seeds at the cotyledonary-embryo stage, respectively.
