# Supplementary figures and images for: Integration of Small RNA and Transcriptome Sequencing Reveal the Roles of miR395 and ATP Sulfurylase in Developing Seeds of Chinese Kale
Source: Front Plant Sci. 2022 Feb 3;12:778848. doi: 10.3389/fpls.2021.778848 (PMC8851238; doi:10.3389/fpls.2021.778848)

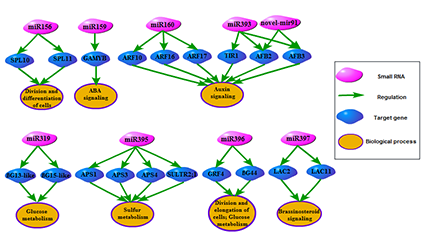

Supplement: Supplementary Figure 1 — Regulatory network of miRNAs and their predicted target genes related to Chinese kale seed development in PC vs. PD, PC vs. SC, PD vs. SD, and SC vs. SD. PC and SC mean sRNA libraries of silique walls and seeds at the torpedo-embryo stage, respectively. PD and SD mean sRNA libraries of silique walls and seeds at the cotyledonary-embryo stage, respectively. [file Image_1.tif]
